# Osteoarthritis Severely Decreases the Elasticity and Hardness of Knee Joint Cartilage: A Nanoindentation Study

**DOI:** 10.3390/jcm8111865

**Published:** 2019-11-03

**Authors:** Adam Aron Mieloch, Magdalena Richter, Tomasz Trzeciak, Michael Giersig, Jakub Dalibor Rybka

**Affiliations:** 1 Center for Advanced Technology, Adam Mickiewicz University in Poznan, Uniwersytetu Poznańskiego 10 Street, 61-614 Poznan, Poland; 2 Faculty of Chemistry, Adam Mickiewicz University in Poznan, Uniwersytetu Poznańskiego 8 Street, 61-614 Poznan, Poland; 3 Department of Orthopedics and Traumatology, Poznan University of Medical Sciences, 28 czerwca 1956r. Street No. 135/147, 61-545 Poznan, Poland; 4 Department of Physics, Institute of Experimental Physics, Freie Universität, Arnimallee 14, 14195 Berlin, Germany

**Keywords:** articular cartilage, osteoarthritis, elastic modulus, mechanical properties, nanoindentation

## Abstract

The nanoindentation method was applied to determine the elastic modulus and hardness of knee articular cartilage. Cartilage samples from both high weight bearing (HWB) and low weight bearing (LWB) femoral condyles were collected from patients diagnosed with osteoarthritis (OA). The mean elastic modulus of HWB cartilage was 4.46 ± 4.44 MPa in comparison to that of the LWB region (9.81 ± 8.88 MPa, *p* < 0.001). Similarly, the hardness was significantly lower in HWB tissue (0.317 ± 0.397 MPa) than in LWB cartilage (0.455 ± 0.434 MPa, *p* < 0.001). When adjusted to patients’ ages, the mean elastic modulus and hardness were both significantly lower in the age group over 70 years (*p* < 0.001). A statistically significant difference in mechanical parameters was also found in grade 3 and 4 OA. This study provides an insight into the nanomechanical properties of the knee articular cartilage and provides a starting point for personalized cartilage grafts that are compatible with the mechanical properties of the native tissue.

## 1. Introduction

Articular cartilage (AC) is a highly specialized weight-bearing tissue that provides low friction during joint articulation. Due to its unique biomechanical functions, AC is mainly avascular, aneural, and alymphatic in structure and is capable of withstanding intensive cyclic loading and shear stress. The molecular composition of the AC perfectly reflects its physiological functions. It is composed of 70–80% water, 15% collagens (predominantly type II collagen), 9% aggrecan, and 3% chondrocytes. The cartilage matrix composition varies depending on the cartilage zone (i.e., tangential, transitional, radial, and calcified). Besides type II collagen, AC contains minute amounts of other types of collagen: III, VI, IX, XI, XII, and XIV [1]. Aggrecan is the main proteoglycan comprising two types of glycosaminoglycans (GAGs): chondroitin sulphate and keratin sulphate. GAGs are highly polyanionic and can bind up to 50 times their weight in water. This mechanism endows AC with its tensile strength, stiffness, and elasticity. The presence of structured collagen fibers and proteoglycans reduces the friction of the articular surface and provides high resistance to mechanical stress, ensuring painless movement in the joints [2]. 

AC damage may occur either as a result of biological factors (e.g., imbalanced expression of cytokines) or mechanical factors [3]. Its avascular structure and low cellular content render AC incapable of efficient self-renewal [4,5]. Mechanical damage or progressing degradation results in numerous morphological, biochemical, and biophysical changes in cartilage structure [6]. Gradual deterioration is also a hallmark of osteoarthritis (OA) [7]. Cartilage erosion is thought to be caused by sustained imbalance between catabolic and anabolic processes. Increased activity of enzymes such as, e.g., matrix metalloproteinases (MMPs) contributes to extracellular matrix (ECM) breakdown. The erosion begins with the truncation of essential components such as collagens and aggrecans [8]. Due to proteoglycan loss, the most superficial cartilage zone (tangential) becomes fibrillated. The progression of matrix degradation results in increased water content and disruption of the collagen network, which, in turn, deteriorates the mechanical properties of the tissue and initiates the compensational synthesis of type II collagen [9]. Further alterations increase cartilage vulnerability to mechanical loads and lead to secondary changes such as subchondral bone sclerosis or osteophyte formation [10]. While the morphological and histological features of OA are well established, the underlying molecular mechanisms are still not completely understood. The mechanical characterization at the nano scale may provide important cues toward unraveling the molecular complexity of the disease. 

Nanoindentation has been developed primarily for the nanomechanical characterization of non-biological surfaces. Paradoxically, limitations of this method stem from its high precision and accuracy. Pronounced heterogeneity of the biological surfaces’ topography renders the utilization of this method quite challenging. Nonetheless, if applied properly, the method offers unmatched accuracy and provides a deep insight into the nanomechanical properties of a given tissue. Although other clinical indentation devices exist, they lack the sensitivity to expose local and distinct changes in the mechanical features of the AC [11].

A study by Stolz et al. inspired us to investigate the potential use of nanoindentation to reveal the discrete changes at the nanoscale occurring during the course of OA [12]. The majority of studies regarding the mechanical properties of AC have been based on intact (healthy) tissue [13,14,15,16]. Only a few experimental studies so far have characterized the properties of degenerated tissue [17,18,19,20,21]. Nia et al. investigated a murine femur cartilage elastic modulus and showed that aggrecan depletion led to a significant decrease in the elastic modulus from 2.0 MPa to around 0.4 MPa [17]. Furthermore, Doyran et al. observed, that in a murine post-traumatic model of OA, changes in cartilage mechanical properties markedly preceded the histological signs of the disease and were detectable at 1 week [22]. Interestingly, when the decrease in the elastic modulus was tested for the case of human osteoarthritic cartilage, it was not correlated with the disease progression [17]. Other studies indicated both an increase and decrease in cartilage elasticity during the course of OA [23,24]. It has been shown that the elastic modulus varies depending on the depth of the indent; therefore, each zone displays slightly different mechanical properties [16]. Similarly, different regions of the knee joint are exposed to different magnitudes of forces. Based on those differences, two types of regions could be distinguished—low weight bearing (LWB) and high weight bearing (HWB). Due to higher exposure to mechanical stress, the HWB region is more prone to the development of OA.

Regarding cartilage repair techniques, the difference between the mechanical properties of the LWB and HWB regions could eventually impact the outcome of AC repair. Differences in the mechanical characteristics between the tissue and a graft may impair its integration. Therefore, whether cell-loaded or cell-free, grafts and scaffolds should represent appropriate mechanical features to support the loading of the joint surfaces and thus easily integrate with the surrounding tissue.

Currently, there are no comprehensive studies on the mechanical properties of high weight bearing and low weight bearing articular cartilage at different stages of OA. This study describes the mechanical features of articular cartilage in terms of the hardness and elastic modulus. A novel implementation of the nanoindentation technique provides an insight into the biomechanical properties of osteoarthritic cartilage.

## 2. Materials and Methods

### 2.1. Samples Collection

Samples of AC were harvested from 75 patients diagnosed with OA undergoing a total knee replacement procedure at the Department of Orthopedics and Traumatology, Poznan University of Medical Sciences. All subjects gave their informed consent for inclusion before they participated in the study. The study was conducted in accordance with the Declaration of Helsinki, and the protocol was approved by the Ethics Committee of Poznan University of Medical Sciences (permission No. 1016/16), and written consent from each patient was obtained.

**Figure 1:**
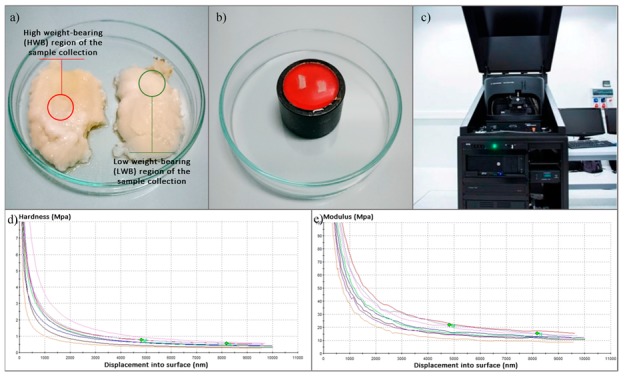
Graphical description of the experiment design: (**a**) indication of locations chosen for sample harvesting; (**b**) sample prepared for measurement; (**c**) nanoindenter used in the study; (**d**,**e**) exemplary data obtained from the measurements.

The OA was diagnosed according to the American College of Rheumatology (ACR) criteria. The exclusion conditions included the presence of rheumatoid arthritis, osteotomy, and post-traumatic osteoarthritis. The radiological stage of the disease was evaluated according to the Kellgren–Lawrence (K-L) scale. The AC specimens were taken from both the medial and the lateral femoral condyle of each patient. Regarding the joint axial deformation (varus or valgus) and tissue morphology, samples were then marked as HWB or LWB (Figure 1). 

### 2.2. Nanoindentation Measurements

**Figure 2:**
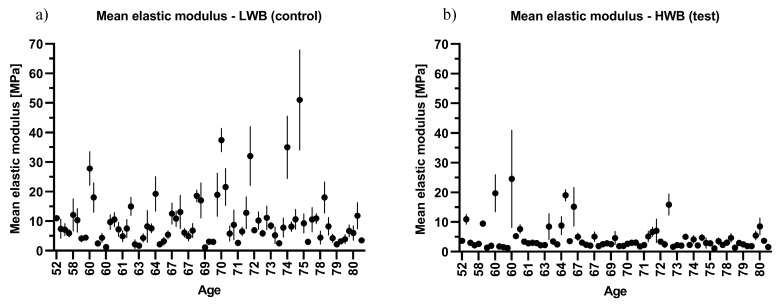
Scatter plot of the mean elastic modulus against age: (**a**) low weight bearing (LWB) sample and (**b**) high weight bearing (HWB) sample.

The cartilage was cut with a surgical blade to obtain samples of at least 3.0 mm × 3.0 mm of flat surface. For the nanoindentation measurement, the AC samples were fixed in acrylic resin (Form Plast, Zhermapol^®^, Warsaw, Poland) to dedicated holders, with the superficial layer facing the indenter. After fixation, samples were rehydrated at RT with phosphate buffer saline (PBS) for 15 min. Before analysis, the excess PBS was poured onto the samples to prevent drying during measurement. The indentation tests were conducted on a nanoindenter Agilent G200 with a DCMII head (Agilent Technologies, Inc., Santa Clara, CA, USA) fitted with a Berkovich-type indenter tip (Figure 2). The area function was calculated according the formula

(1) $$A\left(h_{c}\right)=\;m_{0}+h_{c}^{2}+m_{1}+h_{c}+m_{2}+h_{c}^{\left(\frac{1}{2}\right)}+\cdots +\;m_{n}+h_{c}^{\left(\frac{1}{2^{\left(n-1\right)}}\right)}$$

where the nominal value was *m*_0_ = 24.5.

The tip was calibrated before each sample measurement on quartz crystal (Young’s modulus E = 74 GPa). The measurements were performed in CSM mode (Continuous Stiffness Measurement). Indentations were performed at a depth of up to 10 µm with a strain rate of 1 [1/s] and a Poisson’s ratio of 0.4. In order to calculate the Hardness and Elasticity modulus, determination of the elastic stiffness of the contact is required. Typically, it is derived from the slope of the load–displacement curve during the unload segment [25]. However, this calculation only gives the results for the maximum penetration depth. In our experimental setup, due to the continuous stiffness measurement technique (CSM), the measurement of the elastic stiffness of the contact (and thus the hardness and elasticity modulus) was obtained continuously during the loading. In CSM measurements, the additional harmonic force (with the amplitude in the range of nanometers) is added to the nominally increasing load. The displacement response of the indenter at this harmonic frequency can be analyzed in terms of the displacement amplitude, phase angle, and excitation amplitude. Solving the response equations (described elsewhere [26]) results in the determination of the elastic stiffness of the contact as a continuous function of the depth.

For each sample, 12 indents were performed in a 3 × 4 matrix with 200 μm x,y indent separation. The maximum depth of the indentation was 10.0 μm. The mean elastic modulus and hardness were obtained from the 5.0–8.0 μm indentation depth range. Exemplary raw data obtained from the measurements can be found in the Supplementary Material section (Figures S1–S6).

### 2.3. Histological Analysis

For histology, representative samples of the HWB articular cartilage of patients with K-L grades 2–4 were prepared for the experiments. Cartilage was harvested, fixed in 4% paraformaldehyde, decalcified in 12% EDTA, and embedded in parafin. Serial 5 μm thick sections were cut and stained with Safranin-O/Fast Green (Supplementary Material, Figure S7).

### 2.4. Statistical Analysis

Data were analyzed with Statistica version 13.1 (Tibco Software, Inc., Palo Alto, CA, USA). Descriptive statistics are reported as means, standard deviations (SD), medians, and minimum and maximum values. The Shapiro–Wilk test was used to assess the normality of distributions in the test score. If the data were normally distributed, parametric statistics were used for analyzing the data. The significance of the differences between the results of the tested and control sample was calculated using a paired t-test or non-parametric Wilcoxon signed-ranks test. The non-parametric Mann–Whitney test was conducted to compare the mean elastic modulus and hardness in patients divided by age and sex. The non-parametric Kruskal–Wallis test was used to analyze the differences between the mean elastic modulus and hardness in patients divided by BMI and Kellgren–Lawrence on more than two groups. The Dunn’s post hoc test was used to show the difference between the tested groups. *p*-values of less than 0.05 were considered statistically significant.

## 3. Results

The anthropometric characteristics of the patients are summarized in Table 1. The evaluation of knee radiographs using the K-L grading system showed no patients graded 0, 16 patients graded 2, 39 patients graded 3, and 20 patients graded 4. 

The mean elastic modulus and hardness of HWB cartilage (tested) were significantly lower, when compared to that of LWB (control) (Table 2). The relative frequency values of the mean elastic modulus and hardness obtained in both groups can be found in the Supplementary Materials section (Figures S8 and S9). The difference remained significant when estimated for males and females alone (Table 3). For tested samples, values were higher in females, while they were lower in the control tissue, when compared to males. However, when testing within the HWB and LWB groups of male and female samples, no significant differences were found (Supplementary Material, Table S1: Elastic modulus and hardness of HWB and LWB articular cartilage; adjusted to patients’ sex tested within the groups). 

**Figure 3:**
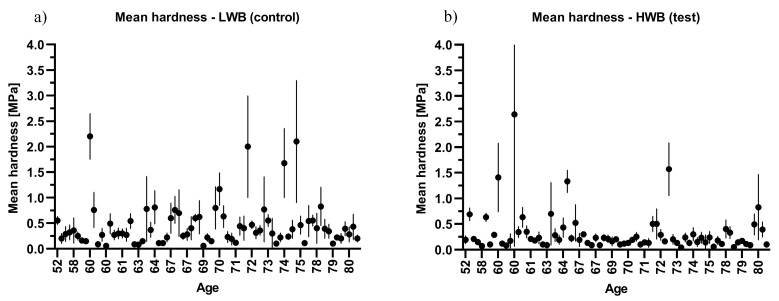
Scatter plot of hardness against age: (**a**) low weight bearing (LWB) sample and (**b**) high weight bearing (HWB) sample.

From the data shown in Table 4, it appears that there is a significant decline in the biomechanical properties of cartilage with increasing age. When adjusted to age, the mean elastic modulus and hardness of articular cartilage were significantly lower in the age group over 70 years (Table 4). These trends are shown in Figure 2; Figure 3, which present the combined sample mean and standard deviation plotted against the patients’ ages. 

The evaluation of the biomechanical properties of articular cartilage in patients at different stages of OA revealed 2–3-fold lower values for grade 3 and 4 HWB sites in terms of the elastic modulus. For hardness, a statistically significant difference was found for grade 3 samples. No appreciable differences were found for grade 2 and 4 OA (Table 5).

However, no statistically significant differences were found when the biomechanical parameters of the cartilage were grouped by patients’ ages, sexes, BMI values, and OA grades and tested within the groups and between the groups (Table 6).

Moreover, increasing age and OA grade were not correlated with the decrease of articular cartilage mechanical properties when analyzed within the HWB and LWB groups (Supplementary Material, Table S2: Correlations between high weight-bearing cartilage (HWB) and low weight-bearing cartilage (LWB) mechanical parameters and age or OA grade tested within the groups).

## 4. Discussion

Tissue engineering techniques permit an innovative approach to articular cartilage repair. However, information about the mechanical properties of human cartilage altered by joint degenerative disease is limited. AC is an inhomogeneous tissue, in which mechanical properties depend mostly on the ECM composition. Its biomechanical function is related to the water content and collagen, proteoglycans, and hyaluronate concentrations and to the interactions between these components. Consequently, alterations in the ECM components and disrupted tissue integrity, either by injury or disease, result in deterioration of the mechanical strength. 

To our knowledge, this is one of the first studies of the nanomechanical properties of weight-bearing and non-weight-bearing articular cartilage at different stages of OA conducted at this scale. Researchers are consistent in the view that the elastic modulus of articular cartilage lies within the range of a few MPa [12,13,17,19,24]. The results of our work are commensurate with the previous studies, where the mechanical properties of articular cartilage were measured. Moshtagh et al. reported the average elastic modulus of the medial tibia plateau cartilage as being 2.6 ± 1.4 MPa, while that of the lateral tibia was reported as being 4.2 ± 2.6 MPa [27]. Antons et al. observed values ranging from 0.020 ± 0.003 MPa in the superficial zone to 6.44 ± 1.02 MPa in the calcified zone of human femoral condyle cartilage [16]. Other researchers have presented contradictory results. Sergerie et al. showed that ECM stiffness decreases along the cartilage zones of the porcine cartilage growth plate, while Park et al. showed that rabbit growth plate stiffness increases across the same region [28,29]. Therefore, site-specific properties, local variation, and cartilage sample thickness should not be neglected [30,31,32]. Observed discrepancies in the values of hardness and elastic modulus could stem from measuring different cartilage zones, which was unavoidable due to inhomogeneous structure of the osteoarthritic cartilage. Moreover, it seems that nanoscale indentation with sharp probes picks up the mechanics of individual macromolecules and is reflected the elasticity of collagen or aggrecan macromolecules [11,33,34]. Chandran et al. stated that an indentation depth of at least 0.6 μm is required to obtain values of matrix elasticity, instead of a rough superficial layer [14]. In the study by Antons et al., the thickness of the samples ranged from 1484 ± 75.23 μm to 3624.4 ± 164.11 μm [16]. In a study by Moshtagh et al., it was observed that each 60 μm change in indenter location could result in a 20-fold variation in the measurement [27].

Values reported by others, suggest, that our measurements were taken in the deep layers of cartilage, rather than in the superficial zone. Additionally, measuring the superficial layer would be very challenging, since the samples were harvested from donors diagnosed with OA. In general, special care was taken to harvest the samples from precisely the same area of the femoral condyle. Nevertheless, the disease stage could influence the accessibility of the tissue, especially from the HWB region of the cartilage. Despite some technical difficulties, our study revealed a significant decrease in the biomechanical properties of the articular cartilage associated with age and disease progression. We observed a 2–3-fold decrease in the elastic modulus and the hardness of cartilage in patients over 70 years of age. Similarly, a significant deterioration of the parameters was observed in stages 2 to 4 of OA. Peters et al. also found that increasing age and OA grade were strongly correlated with a decrease in cartilage shear modulus (*p* = 0.003 and *p* = 0.007, respectively) [20]. Cao et al. noticed that the decreased stiffness of the OA cartilage may be caused by increased water content and elevated permeability as well as to a decreased proteoglycans content [13]. Nia et al. found that aggrecan depletion from a mouse femur led to a significant decrease in the elastic modulus (from approx. 2.0 to 0.4 MPa). However, the aggrecan depletion was not observed for human cartilage in correlation to the progression of OA [17].

In spite of the intriguing results, there were several limitations in our study. First of all, a biochemical analysis was not performed to elucidate the cartilage composition and its correlation with the mechanical properties of the tissue. Another limitation is the lack of an appropriate reference material, such as a cartilage sample from a healthy donor, which may have provided comparative values of measured parameters and the extent of the impact of OA on the articular cartilage biomechanics. This, however, could be done in the future using cadaver cartilage taken from young subjects. Moreover, the indenter tip used in this study (Berkovich type) could influence the materials’ behavior under indentation. This is related to the size of the indentation region, which is different in cases of Berkovich and conical tips [35]. However, for biological samples, a spherical indentation tip is more commonly used, due to its more favorable geometry, providing a good alignment between the indenter and the material surface [36,37].

## 5. Conclusions

This study demonstrates the regional mechanical properties of the articular cartilage of the knee joint taken from a representative number of OA patients. The results provide insight into the mechanical behavior of the cartilage at different stages of OA in correlation to the patients’ ages, which is essential from the clinical perspective. Matching the mechanical characteristics of the tissue and graft is crucially important for proper integration with the surrounding tissue [38,39,40,41]. Currently, there are no methods to select an ideal biomaterial-based graft for repairing cartilage lesions. Moreover, the repair procedures are mostly performed on the high weight-bearing surface of the femoral condyle, thus indicating the importance of assessing the biomechanical properties of surrounding tissue prior to graft implantation. This would significantly raise the chances for successful and long-term clinical improvement of the operated patients. 

## Figures and Tables

**Table 1:** Baseline demographics of the patients.

Variable	Mean ± SD	Median	Min–Max
Age (years)	68.5 ± 7.5	69.0	52.0–82.0
Weight (kg)	84.0 ± 14.7	82.0	55.0–118.0
Height (cm)	163.8 ± 8.7	164.0	146.0–183.0
BMI (kg/m^2^)	31.3 ± 5.0	31.0	22.0–46.1

BMI, body mass index; SD, standard deviation.

**Table 2:** Elastic modulus and hardness of high weight bearing (HWB) and low weight bearing (LWB) articular cartilage.

	HWB Cartilage			LWB Cartilage			
Variable	Mean ± SD	Median	Min–Max	Mean ± SD	Median	Min–Max	*p*-Value
Elastic modulus (MPa)	4.46 ± 4.44	2.90	1.10–24.35	9.81 ± 8.88	7.40	1.10–51.00	<0.001 *
Hardness (MPa)	0.317 ± 0.397	0.190	0.040–2.640	0.455 ± 0.434	0.320	0.060–2.200	<0.001 *

* Wilcoxon signed-rank test.

**Table 3:** Elastic modulus and hardness of high weight bearing (HWB) and low weight bearing (LWB) articular cartilage adjusted to patients’ sexes.

			HWB Cartilage			LWB Cartilage			
Variable	Sex	*n*	Mean ± SD	Median	Min–Max	Mean ± SD	Median	Min–Max	*p*-Value
Elastic modulus (MPa)	Female	57	4.52 ± 4.17	2.94	1.10–19.70	9.54 ± 8.56	7.11	1.10–51.00	<0.001 *
	Male	18	4.25 ± 5.34	2.60	1.30–24.35	10.66 ± 10.05	8.30	1.19–37.40	0.002 *
Hardness (MPa)	Female	57	0.309 ± 0.318	0.200	0.040–1.570	0.442 ± 0.424	0.320	0.060–2.200	0.004 *
	Male	18	0.343 ± 0.594	0.130	0.070–2.640	0.493 ± 0.478	0.330	0.060–2.000	0.022 *

* Wilcoxon signed-rank test.

**Table 4:** Elastic modulus and hardness of high weight bearing (HWB) and low weight bearing (LWB) articular cartilage adjusted to patients’ ages.

			HWB Cartilage			LWB Cartilage			
Variable	Age	*n*	Mean ± SD	Median	Min–Max	Mean ± SD	Median	Min–Max	*p*-Value
Elastic modulus (MPa)	<69	39	5.28 ± 5.50	2.94	1.22–24.35	8.24 ± 5.94	7.11	1.10–27.80	0.002 *
	>70	36	3.56 ± 2.71	2.83	1.10–15.86	11.51 ± 11.07	8.18	2.13–51.00	<0.001 *
Hardness (MPa)	<69	39	0.371 ± 0.480	0.210	0.070–2.640	0.389 ± 0.372	0.280	0.060–2.00	0.085 *
	>70	36	0.259 ± 0.277	0.165	0.040–1.570	0.525 ± 0.489	0.390	0.100–2.100	<0.001 *

* Wilcoxon signed-rank test.

**Table 5:** Elastic modulus and hardness of high weight bearing (HWB) and low weight bearing (LWB) articular cartilage adjusted to the Kellgren–Lawrence (K-L) osteoarthritis (OA) grading system.

			HWB Cartilage			LWB Cartilage			
Variable	K-L Grade	*n*	Mean ± SD	Median	Min–Max	Mean ± SD	Median	Min–Max	*p*-Value
Elastic modulus (MPa)	2	16	5.84 ± 5.04	3.76	1.70–18.99	11.32 ± 8.87	8.89	1.10–34.96	0.073
	3	39	3.78 ± 3.53	2.75	1.22–19.70	10.29 ± 9.90	8.13	1.67–51.00	<0.001 *
	4	20	4.66 ± 5.42	2.59	1.10–24.35	7.66 ± 6.45	6.86	1.19–32.00	0.008 *
Hardness (MPa)	2	16	0.416 ± 0.438	0.240	0.070–1.570	0.463 ± 0.391	0.370	0.060–1.680	0.772
	3	39	0.248 ± 0.230	0.190	0.050–1.410	0.481 ± 0.462	0.370	0.080–2.200	<0.001 *
	4	20	0.375 ± 0.579	0.165	0.040–2.640	0.396 ± 0.426	0.285	0.060–2.000	0.179 *

Paired *t*-test, * Wilcoxon signed-rank test.

**Table 6:** Differences between the values of elastic modulus and hardness of high weight bearing (HWB) and low weight bearing (LWB) articular cartilage adjusted to age, sex, BMI, and the K-L OA grade tested within the groups and between the groups.

		HWB Cartilage		LWB Cartilage		HWB vs. LWB	
		Elastic Modulus	Hardness	Elastic Modulus	Hardness	Elastic Modulus	Hardness
Age	<69	0.311 *	0.270 *	0.270 *	0.127 *	0.124 *	0.160 *
	>70						
Sex	f	0.283 *	0.208 *	0.941 *	0.936 *	0.519 *	0.355 *
	m						
BMI	normal	0.548 **	0.406 **	0.742 **	0.738 **	0.938 **	0.991 **
	overweight						
	obese						
K-L grade	2	0.130 **	0.365 **	0.414 **	0.482 **	0.523 **	0.688 **
	3						
	4						

* Mann–Whitney U test, ** Kruskal–Wallis test.

## Supplementary Materials

The following are available online at https://www.mdpi.com/2077-0383/8/11/1865/s1, Figure S1: Exemplary raw data obtained from the measurements; Figure S2: Exemplary raw data obtained from the measurements; Figure S3: Exemplary raw data obtained from the measurements; Figure S4: Exemplary raw data obtained from the measurements; Figure S5: Exemplary raw data obtained from the measurements; Figure S6: Exemplary raw data obtained from the measurements; Figure S7: Representative histology sections of HWB cartilage (Safranin-O/Fast Green staining); Figure S8: Relative frequency of mean elastic modulus of: (a) low weight bearing (LWB) sample and (b) high weight bearing (HWB) sample; Figure S9: Relative frequency of mean hardness of: (a) low weight bearing (LWB) sample and (b) high weight bearing (HWB) sample. Table S1: Elastic modulus and hardness of high weight bearing (HWB) and low weight bearing (LWB) articular cartilage; adjusted to patients’ sex tested within the groups, Table S2: Correlations between high weight bearing cartilage (HWB) and low weight bearing cartilage (LWB) mechanical parameters and age or OA grade tested within the groups.
