# Supplementary material for: Osteoarthritis Severely Decreases the Elasticity and Hardness of Knee Joint Cartilage: A Nanoindentation Study
Source: J Clin Med. 2019 Nov 3;8(11):1865. doi: 10.3390/jcm8111865 (PMC6912408; doi:10.3390/jcm8111865)
Supplement: Supplementary file 1 [file jcm-08-01865-s001.pdf]

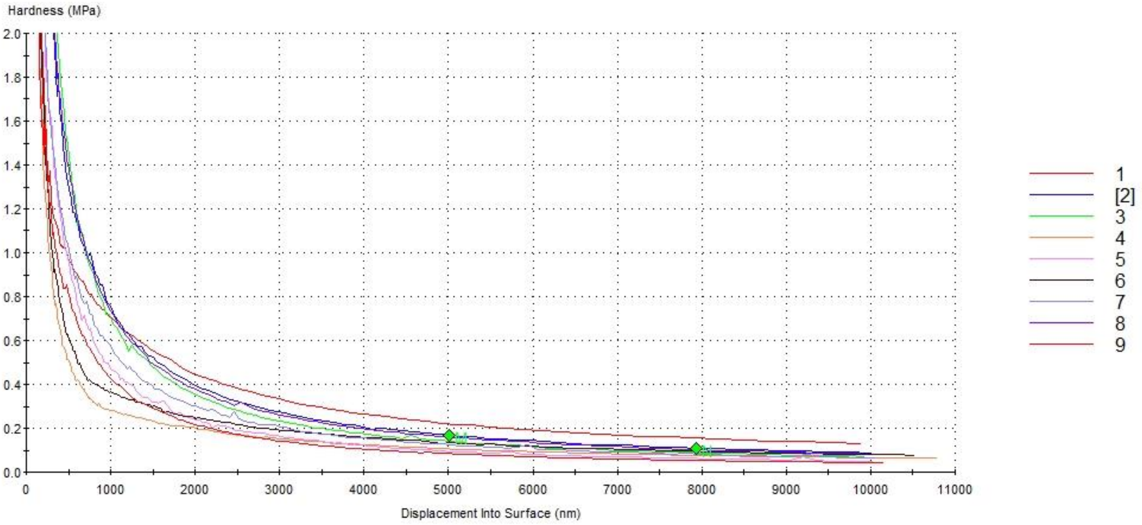

Figure S1. Exemplary raw data obtained from the measurements.

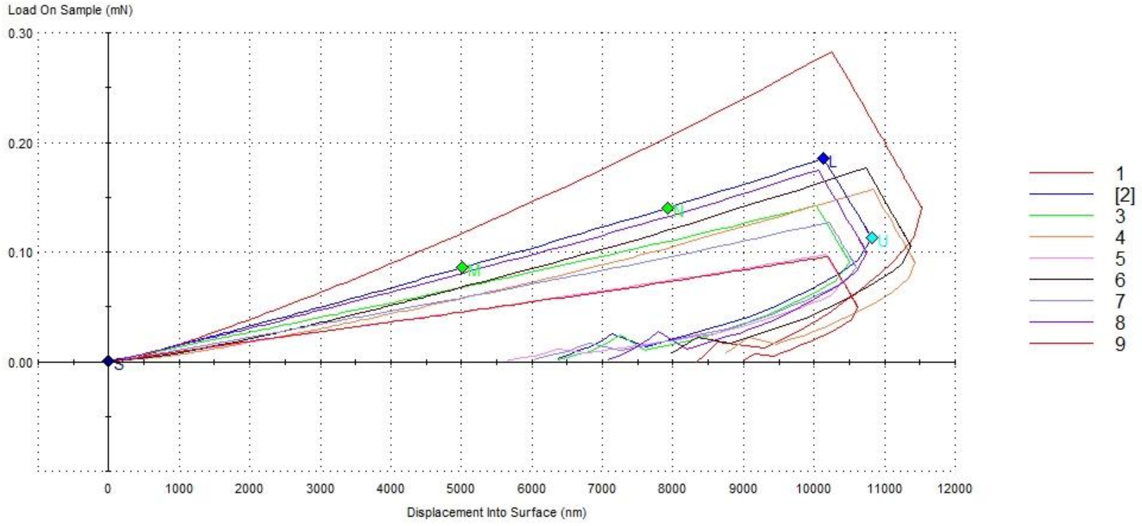

Figure S2. Exemplary raw data obtained from the measurements.

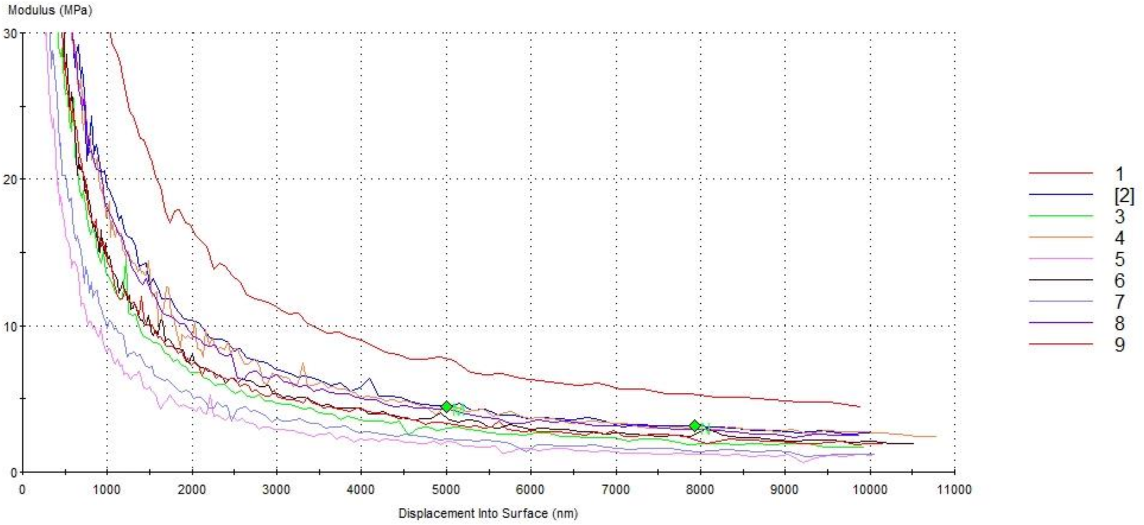

Figure S3. Exemplary raw data obtained from the measurements.

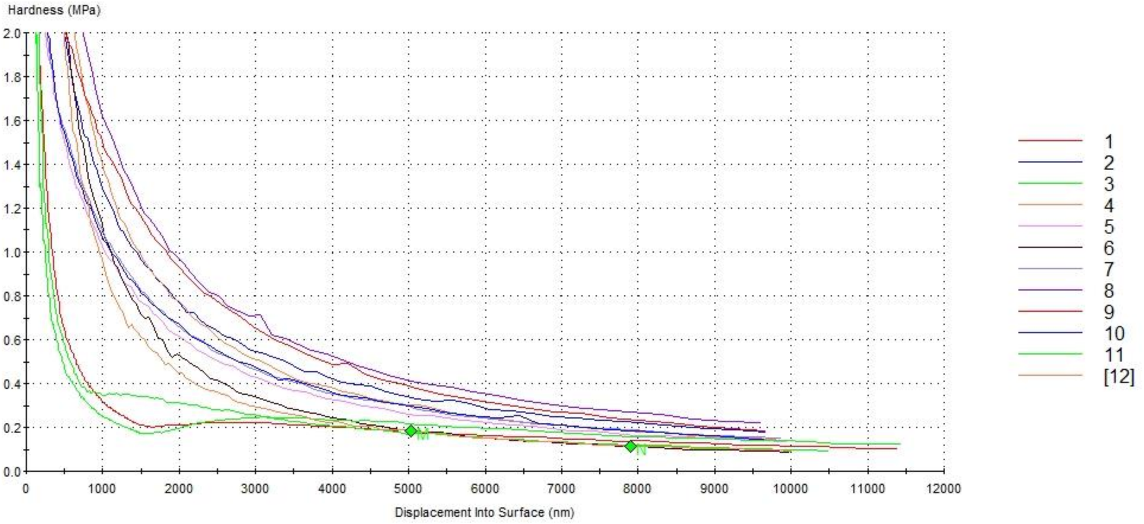

Figure S4. Exemplary raw data obtained from the measurements.

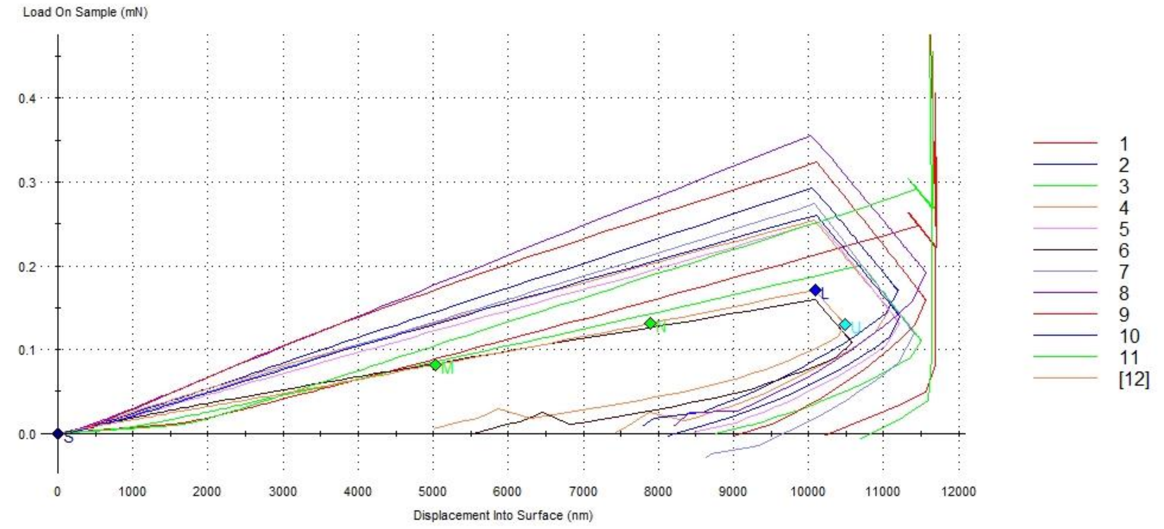

Figure S5. Exemplary raw data obtained from the measurements.

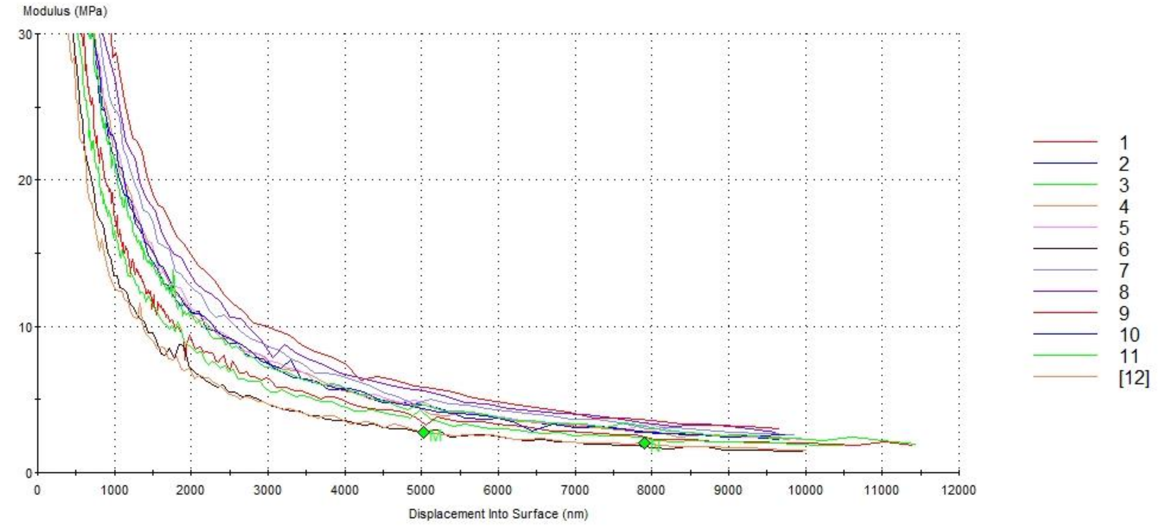

Figure S6. Exemplary raw data obtained from the measurements.

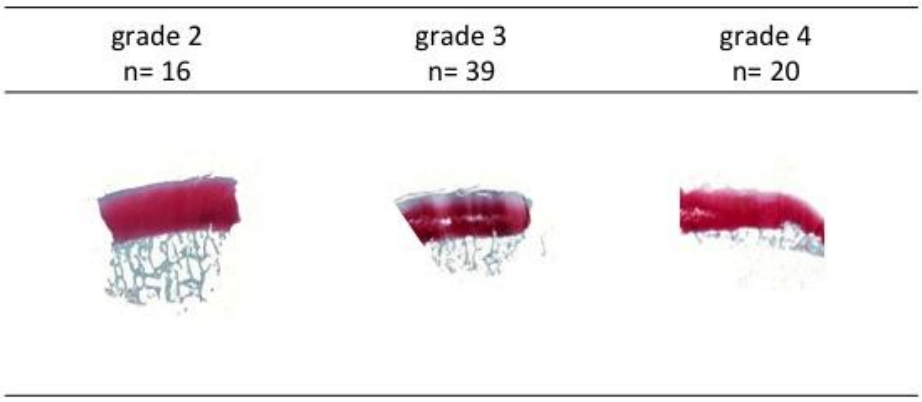

Figure S7. Representative histology sections of HWB cartilage (Safranin-O/Fast Green staining).

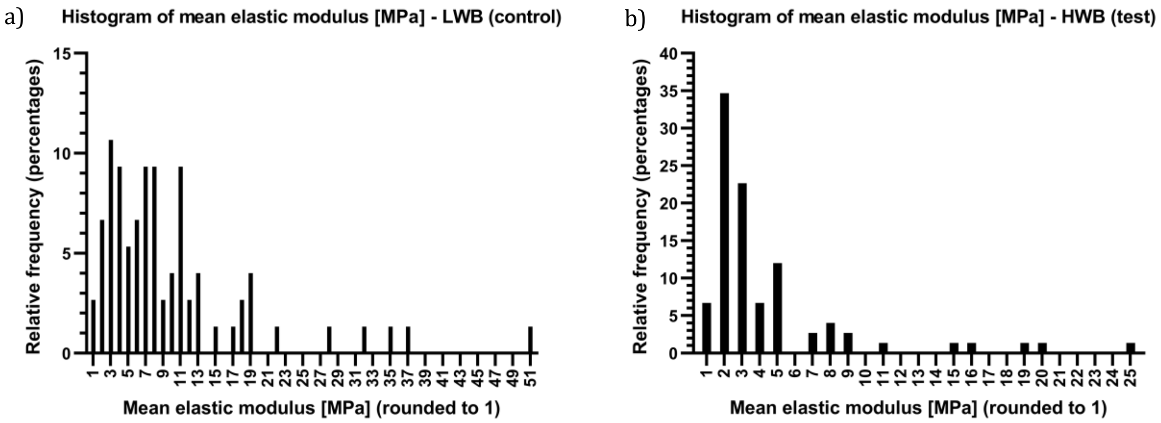

Figure S8. Relative frequency of mean elastic modulus of: (**a**) low weight bearing (LWB) sample and (**b**) high weight bearing (HWB) sample.

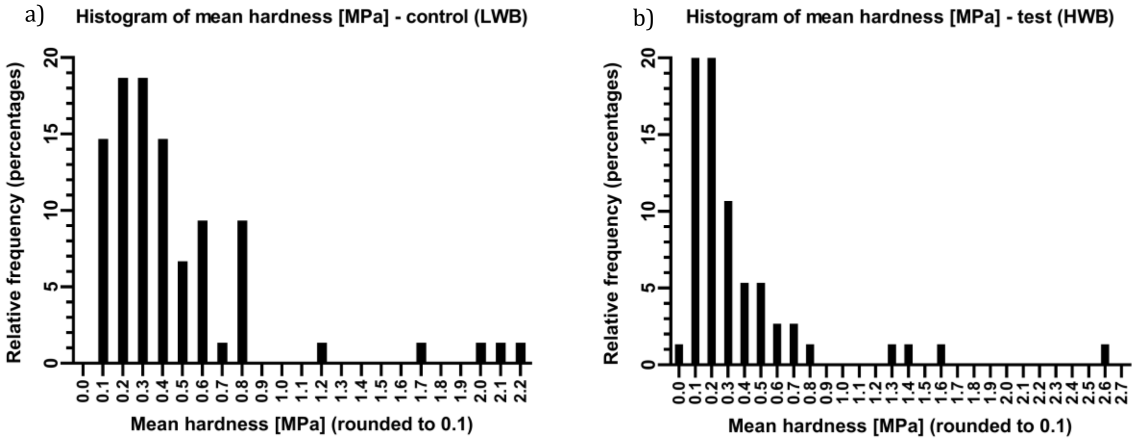

Figure S9. Relative frequency of mean hardness of: (**a**) low weight bearing (LWB) sample and (**b**) high weight bearing (HWB) sample.

Table S1. Elastic modulus and hardness of high weight bearing (HWB) and low weight bearing (LWB) articular cartilage; adjusted to patients' sex tested within the groups.

	Males			Females			
**Variable**	**Mean ± SD**	**median**	**min-max**	**Mean ± SD**	**median**	**min-max**	***p*-value**
**HWB cartilage Elastic modulus (MPa)**	4.25 ± 5.34	2.60	1.30–24.35	4.52 ± 4.17	2.94	1.10–19.70	0.283
**LWB cartilage Elastic modulus (MPa)**	10.66 ± 10.05	8.30	1.19–37.40	9.54 ± 8.56	7.11	1.10–51.00	0.941
**HWB cartilage Hardness (MPa)**	0.343 ± 0.594	0.130	0.070–2.640	0.309 ± 0.318	0.200	0.040–1.570	0.208
**LWB cartilage Hardness (MPa)**	0.493 ± 0.478	0.330	0.060–2.000	0.442 ± 0.424	0.320	0.060–2.200	0.936

Mann-Whitney U test.

Table S2. Correlations between high weight bearing cartilage (HWB) and low weight bearing cartilage (LWB) mechanical parameters and age or OA grade tested within the groups.

**Parameters**	**R Spearman**	***p*-value**
HWB cartilage Elastic Modulus & age	-0,119	0,308
HWB cartilage Elastic Modulus & K-L	-0,200	0,085
LWB cartilage Elastic Modulus & age	0,012	0,921
LWB cartilage Elastic Modulus & K-L	-0,153	0,190
HWB cartilage Hardness & age	-0,139	0,233
HWB cartilage Hardness & K-L	-0,140	0,232
LWB cartilage Hardness & age	0,125	0,286
LWB cartilage Hardness & K-L	-0,123	0,293
HWB vs LWB Elastic Modulus & age	0,085	0,468
HWB vs LWB Elastic Modulus & K-L	-0,110	0,348
HWB vs LWB Hardness & age	0,131	0,262
HWB vs LWB Hardness & K-L	-0,006	0,962
